# Determination of Acrylamide in Biscuits by High-Resolution Orbitrap Mass Spectrometry: A Novel Application

**DOI:** 10.3390/foods8120597

**Published:** 2019-11-20

**Authors:** Cristiana L. Fernandes, Daniel O. Carvalho, Luis F. Guido

**Affiliations:** REQUIMTE—Departamento de Química e Bioquímica, Faculdade de Ciências, Universidade do Porto, Rua do Campo Alegre 687, 4169-007 Porto, Portugal; cris.rlf@gmail.com (C.L.F.); daniel.carvalho@fc.up.pt (D.O.C.)

**Keywords:** acrylamide, biscuits, mitigation measures, benchmark levels, contaminant

## Abstract

Acrylamide (AA), a molecule which potentially increases the risk of developing cancer, is easily formed in food rich in carbohydrates, such as biscuits, wafers, and breakfast cereals, at temperatures above 120 °C. Thus, the need to detect and quantify the AA content in processed foodstuffs is eminent, in order to delineate the limits and mitigation strategies. This work reports the development and validation of a high-resolution mass spectrometry-based methodology for identification and quantification of AA in specific food matrices of biscuits, by using LC-MS with electrospray ionization and Orbitrap as the mass analyser. The developed analytical method showed good repeatability (RSD_r_ 11.1%) and 3.55 and 11.8 μg kg^−1^ as limit of detection (LOD) and limit of quantification (LOQ), respectively. The choice of multiplexed targeted-SIM mode (t-SIM) for AA and AA-d3 isolated ions provided enhanced detection sensitivity, as demonstrated in this work. Statistical processing of data was performed in order to compare the AA levels with several production parameters, such as time/cooking temperature, placement on the cooking conveyor belt, color, and moisture for different biscuits. The composition of the raw materials was statistically the most correlated factor with the AA content when all samples are considered. The statistical treatment presented herein enables an important prediction of factors influencing AA formation in biscuits contributing to putting in place effective mitigation strategies.

## 1. Introduction

Once Tareke et al. [1] have reported acrylamide (AA) as a carcinogen formed in heated foodstuffs in the food industry, Member States of the European Union and the European Commission have made considerable efforts to investigate AA formation pathways in order to reduce the levels of this compound in processed foods. In addition to being present in foods, AA has also been found in the environment (due to industrial discharges), cosmetics, drinking water, as well as tobacco smoke. Human exposure to AA may be by ingestion, inhalation, or contact with the skin [2]. Dietary exposure is the most concerning, since acrylamide is present in a wide range of everyday foods. Between 10% and 50% of AA of the diet of pregnant women passes through the placenta and breast milk also contains this compound [3]. In the US, most exposure to AA comes from potato chips, breads, cereals, crackers, and other snacks [4]. In Europe, toasted bread, coffee, and potatoes are the main food sources of AA [5].

Human exposure to AA may have toxicological effects (neurotoxicity, genotoxicity, carcinogenicity, and reproductive toxicity), and AA has been classified as carcinogenic by the International Agency for Research on Cancer [6] in the 2A group (probably carcinogenic in humans). AA has an α, β-unsaturated carbonyl group with electrophilic reactivity, which can react with nucleophilic groups of biological molecules, thus contributing to toxic effects. The reaction of AA with proteins is extensive and the products of this reaction are used as biomarkers of its presence [5]. It is metabolized together with glutathione (GSH) and also by epoxidation, resulting in glycidamide (GA). The formation of GA is mediated preferentially by cytochrome P450, and is on the basis of neuro and genotoxicity of AA. Covalent DNA adducts of GA were observed in vitro and in animal experiments and were used as biomarkers [2,5]. 

Only the legal limit of AA for water has been established, with the value of 0.1 μg L^−1^ [7]. The levels of AA in foodstuffs of the Member States of the European Union were monitored between 2007 and 2012. Based on the results, the European Commission outlined indicative values for AA in different foodstuffs [8]. According to Regulation 2017/2158, these values are not safety values but rather indicative values, so that further research is promoted in foods with higher AA levels and consequent reduction throughout the agronomical factors, the food recipe, processing, and final preparation [5].

The level of free asparagine in cereals has been claimed to be the major influence on the formation of AA [9], since the largest pathway of AA formation involves this amino acid. The choice of cereal varieties with lower levels of free asparagine is recommended, but challenging given the influence of environmental conditions on their production [10].

Corn and rice products tend to have lower AA contents than wheat, barley, oats, or rye. Products with whole flours have higher levels of AA [10]. The choice of different varieties of cereals also determines the development of AA: Five varieties of rye with different fertilizations were used to study the effect of nitrogen and sulfur on AA formation. A positive correlation was found between asparagine concentration in grains and the highest levels of nitrogen used and the final concentration of AA [11,12].

The influence of cereal types on bread was investigated by Przygodzka et al. [13], concluding that rye loaves form more AA in cooking, followed by spelled loaves and loaves of refined flour—“white bread.” In the same study, the extraction rates of the flour were compared with AA formation: 100% whole flours obtained higher concentrations of AA, followed by flours with extractions of 70%, indicating that “whole flours” have more AA precursors.

Several AA mitigation measures have been established that involve the use of the enzyme asparaginase, which converts asparagine to aspartic acid, although control of adverse effects on organoleptic properties is necessary [14].

The requirement for ultra-trace level detection of AA has led to the development of several analytical methods, most of which involve chromatographic separation techniques, both liquid and gas chromatography. Determination of AA in food by GC-MS methods can be carried out with or without derivatization. The advantage of derivatization processes is increased volatility and improved selectivity. The bromination [1,15,16,17,18,19,20], xanthydrol [21,22,23,24,25], and silylation [26,27] have been widely used for determining of AA in foodstuffs.

In recent years, the use of ultra-performance liquid chromatography (UPLC) has become more popular because of its high sensitivity and selectivity, without the need for derivatization. Liquid chromatography coupled to mass spectrometry have become the method of choice for the determination of AA in food products, by using different mass analysers. Conventional triple quadrupole (QqQ) have been for long the technique of choice by selecting the characteristic transitions *m*/*z* 72→55, and 72→27. Ion trap [28,29,30] and TOF [28,31,32,33] have also been useful for quantitative analyses of AA.

Considering that the capabilities of high-resolution mass spectrometry (HRMS) based methodologies for quantitative LC/MS analysis of AA in foodstuffs have been scarcely explored [34,35,36] the present work aims at validating a HRMS methodology for detection and quantification of AA in biscuits. The implemented procedure has been applied for investigating the impact of several production parameters on the AA content in biscuits.

## 2. Materials and Methods

### 2.1. Chemicals

Acrylamide (≥ 95% for HPLC) and acrylamide-d3 standard solution (1000 mg L^−1^ in acetonitrile) were purchased from Sigma-Aldrich (Steinheim, Germany). Methanol (for UHPLC), ethanol (99.5%), and dichloromethane (for HPLC) were from Panreac (Barcelona, Spain). High-purity water from a Millipore Simplicity 185 water purification system (Millipore Iberian S. A., Madrid, Spain) was used for all chemical analyses and glassware washing. The solvents employed for HPLC were filtered through a Nylon filter of 0.45 μm pore size (Whatman, Clifton, NJ, USA) and degasified for 10 min in an ultrasound bath.

### 2.2. Standard Solutions

Concentrated stock solutions of acrylamide (1 mg mL^−1^) and acrylamide-d3 (0.5 mg mL^−1^), used as internal standard, were prepared by dissolving the compounds in ethanol. Diluted standard solutions were further prepared by adding the appropriate volume of each stock solution to water.

### 2.3. Biscuit Samples

**Figure 1:**
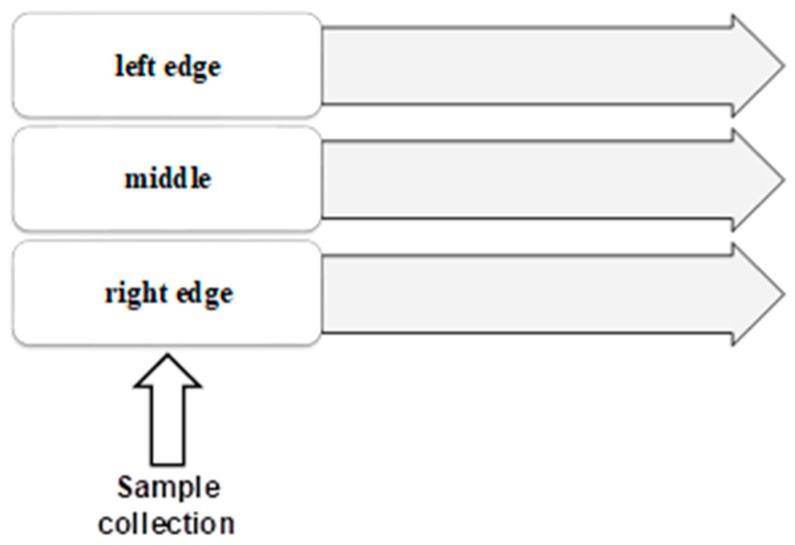
Sample collection points from the baking oven.

This work has been carried out in close collaboration with a leading company at the national level and with an international dimension, whose confidentiality will be maintained for obvious reasons. Four biscuit types were supplied (hereinafter referred to A, B, C, and D), collected from three different sample points in the baking oven, as depicted in Figure 1. Biscuits A, B, and C are made from wheat flour type 65 whereas Biscuit D is made from wheat bran. In addition, Biscuit C has cocoa in its composition.

### 2.4. Sample Preparation

Between three and five biscuits were pooled and grinded in a solid sample grinder (Moulinex, France) and put through an Endecott’s test sieve (London, England). Approximately 1 g of each ground homogenous sample were transferred into a 50 mL polypropylene graduated conical tube with cap. 250 ng of internal standard (acrylamide-d3) and then 15 mL of ultrapure water were added to each tube, which was placed in the ultrasonic bath for 15 min. 2 mL of dichloromethane was added to each tube, left on the rotary shaker for further 20 min. The tubes were centrifuged at 5000 rpm for 15 min. 1500 μL of supernatant from each tube was withdrawn for extraction and purification by solid phase extraction (SPE).

For the SPE clean-up, the Oasis HLB SPE cartridge (6 mL, 200 mg, 30 µm particle size from Waters) was conditioned under vacuum with methanol (3.5 mL), and equilibrated with water (3.5 mL). Then, 1.5 mL of the withdrawn supernatant were loaded on the Oasis HLB SPE cartridge and allowed to pass completely through the sorbent material. The cartridge was rinsed with 500 μL of ultrapure water and samples were eluted with 1.5 mL of water.

For the second step of the clean-up, the Bond Elut AccuCAT SPE cartridge (3 mL, 200 mg, 50 µm particle size from Agilent Technologies) was conditioned under vacuum with methanol (2.5 mL), and equilibrated with water (2.5 mL). Then, the cartridge was loaded with the solution from the previous step and 1 mL was discarded. The remaining volume was collected directly to an injection vial.

### 2.5. LC-ESI-Orbitrap

The samples were separated on Accela HPLC (Thermo Fischer Scientific, Bremen, Germany) Electrospray Orbitrap, using a C18 Phenomenex Germini (Phenomenex, USA), particle size of three microns and size 4.6 mm ID × 150 mm. The samples were eluted through a gradient of 90% solvent A (0.1% HCOOH in water) and 10% solvent B (methanol) for 2 min at a flow rate of 0.4 mL/min, thereafter for 18 min over 100% solvent B and 10 min in a 10% solvent B gradient.

The analysis was performed on a hybrid mass spectrometer LTQ XL OrbitrapTM (Thermo Fischer Scientific, Bremen, Germany), controlled by LTQ Tune Plus Xcalibur 2.5.5 and 2.1.0. The following ionisation (positive mode) parameters were applied: Electrospray voltage 3.2 kV, capillary temperature 300 °C, sheath gas (N_2_), 40 arbitrary units (arb), auxiliary gas (N_2_) 10 (arb), and S-Lens RF level at 25 (arb). The automatic gain control was used to fill the C-trap and gain accuracy in mass measurements (ultimate mass accuracy mode, 1 × 10^5^ ions), the SIM maximum IT was set to 50 ms, the number of micro-scans to be performed was set at three. Mass spectra were recorded in multiplexed targeted-SIM mode (t-SIM) with a mass resolving power of 60,000 full width at half maximum (FWHM) with a quadrupole isolation window of 1.0 Da for isolated ions (72.0444 Da for acrylamide and 75.0632 Da for acrylamide-d3). Chromatograms for a biscuit sample, indicating the acrylamide (AA) and deuterated acrylamide (acrylamide-d3) retention times, are shown in Figure S1.

### 2.6. Analysis of Colour

The biscuits’ colour (Biscuit D) was analyzed with the Minolta CR-410 colorimeter. The parameters used were Luminosity (*L*), Red (*a*), and Yellow (*b*). The biscuits were analyzed in their form of consumption (without being ground), so that the color could be considered a method of control in future industrial tests and quality parameters.

### 2.7. Moisture Content Determination

The biscuits’ moisture level (Biscuit D) was assessed on the same day of the AA extraction. About 5 g of ground biscuit were dried for 3 h at 100 °C. After drying and cooling, the dry mass of the biscuit was measured and the moisture content was calculated.

### 2.8. Statistical Analysis

To measure the strength of relationship between the measured variables, Pearson’s correlation coefficient (*r*) and Spearman’s correlation coefficient (*ρ*) were calculated. While the Pearson correlation coefficient reflects the strength of linear relationships, the Spearman rank correlation reflects the strength of monotonic relationship [37].

The statistical package StatBox 7.5 (Grimmer Logiciel, Paris, France) was used for all statistical calculations.

## 3. Results and Discussion

### 3.1. Method Performance

Limits of detection and quantification (LOD and LOQ) were estimated by using the signal-to-noise method, as specified in the European Pharmacopoeia [38]. The peak-to-peak noise around the AA (*m*/*z* 72.0444) retention time was measured, and subsequently, the concentration of the AA that yielded a signal equal to a certain value of noise to signal ratio was estimated, by comparing measured signals from samples with known low concentrations of the AA with those of blank samples. This method allows a decrease of the signal (peak height) to be observed to the extent that the concentration is reduced through a series of dilutions, establishing the minimum concentration at which the analyte can be reliably quantified. The signal-to-noise (S/N) ratios accepted as estimates of the LOD and LOQ were 3:1 and 10:1, respectively [39]. The values found in this study, based on three measurements of Biscuit A, are 3.55 μg kg^−1^ for LOD and 11.8 μg kg^−1^ for LOQ, as shown in Table 1.

Commission regulation of 20 November 2017 states that the method of analysis used for the analysis of AA must comply with the following criteria: LOQ less than or equal to two fifths of the benchmark level (for benchmark level <125 μg kg^−1^) and less than or equal to 50 μg kg^−1^ (for benchmark level ≥125 μg kg^−1^); LOD less than or equal to three tenths of LOQ [40]. According to the same regulation, the benchmark level for the presence of AA in biscuits and wafers is 350 μg kg^−1^. This means that LOD and LOQ is required to be less than or equal to 15 and 50 μg kg^−1^, respectively. The method herein presented clearly meets these requirements. Moreover, in a proficiency test recently organized by the EURL-PAH, for the determination of the AA content in potato chips, the method performance LOD and LOQ were reported [41]. Twenty-six laboratories guarantee the determination of AA with an average LOD of 22.5 μg kg^−1^ and LOQ 55.8 μg kg^−1^ by liquid chromatography (LC) coupled with mass spectrometry (MS; MS/MS). Nine laboratories participating in this proficiency test reported 15.2 and 36.3 μg kg^−1^ as average LOD and LOQ, respectively, based on GC-MS methods. By using the analytical method herein reported, it is possible to increase the detectability and thus achieve lower limit of quantification, which can be particularly useful in the case of low-abundance AA matrices. The selection of the acquisition mode in the Orbitrap has a direct impact on the detection sensitivity. In a recent paper, Kaufmann demonstrated that the sensitivity of eight selected analytes is strongly increased by the use of SIM (selected ion monitoring) relatively to the FS (full scan) mode (1.5-fold increase for analytes in pure standard solutions and 2-fold increase for analytes spiked in a heavy matrix) [42]. A detailed study of the acquisition method for determination of eight synthetic hormones in animal urine concluded that reducing the scan range for Full MS (using the quadrupole) and targeted modes give higher S/N ratios and thereby better detection limits for analytes in complex matrices [43]. In fact, the targeted-SIM (t-SIM) is not more selective than full MS, but it does provide enhanced detection sensitivity. As only a small fraction of the continuously entering ion beam is sampled by the C-trap, the number of ions transmitted is greatly reduced and a much longer segment of the ion beam can be collected. Accordingly, significantly higher sensitivity can be achieved, mainly for small molecules applications, such as the present case of AA.

The precision of the method was evaluated by measuring the repeatability (intra-day variability). The relative standard deviation was calculated for repeatability (RSDr) by performing eight repeated analyses for samples of the same biscuit. The results showed that the RSDr (11.1%) was less than 12% for a sample with an average AA content of 297.9 μg kg^−1^ (Table 1). The use of isotopically labeled internal standard (acrylamide-d3) is herein especially useful, as sample loss may occur during sample preparation steps prior to analysis, as it is known that the fat/water distribution of the matrix may affect the extraction and analysis.

### 3.2. Acrylamide Content in Biscuits

The optimized and validated procedure was applied to different samples of biscuits collected from the baking oven. Three sample points were considered, as depicted in Figure 1. One is in the middle of the oven and two are in the edges of the oven (left edges and right edges).

**Figure 2:**
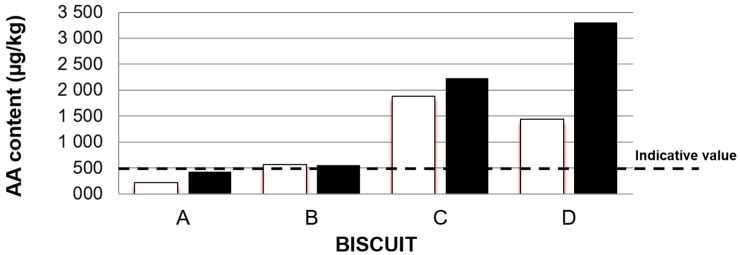
Effect of the position in the oven on the acrylamide content of biscuits. The dashed line depicts the indicative level (500 µg kg^−1^) reported by the European Food Safe Authority [8]. □ edges of the baking oven ■ middle of the baking oven.

A considerable difference was observed between samples collected from the middle and edges of the oven (Table 2). Except for Biscuit B, the AA content is higher for samples taken from the middle of the oven, where the temperatures are higher. The observed increase is higher for Biscuits D (from 1443 up to 3303 μg kg^−1^, corresponding to 129% increase) and A (from 216 up to 431 μg kg^−1^, corresponding to 99% increase). Except for Biscuit A, the AA content found for all the inspected biscuits was above the benchmark level referred to in the European Union (EU) Commission Regulation [40]. Of more concern is the fact that Biscuits C and D contain AA in concentration clearly above (average 2056 and 2373 μg kg^−1^, respectively) the indicative value reported by European Food Safe Authority (dashed line in Figure 2), confirming the pressure of establishing mitigation measures for the reduction of the presence of AA in these matrices.

The current analyses are in line with the hypothesis that the raw materials are the major factors influencing the formation of AA, in particular the asparagine content of cereal flours [44,45,46,47]. The highest value obtained for Biscuit D (average 2373 μg kg^−1^) can be justified by its composition, since one of its raw materials is the wheat bran. Wheat bran is the outer part of the wheat grain, which is removed in flours such as wheat flour type 65. “Whole” flour contains wheat bran and are associated with higher concentrations of asparagine (691 mg kg^−1^) compared to wheat flour type 65 (54.5 mg kg^−1^). Another type of raw material that may increase the concentration of AA in biscuits is that undergoing heat treatment, such as cocoa. Cocoa, which is a raw material with thermal pretreatment and therefore prone to the formation of AA [48], is present in Biscuit C explaining the high content found (average 2056 μg kg^−1^).

### 3.3. Correlation between Acrylamide Content and Biscuit Colour

**Figure 3:**
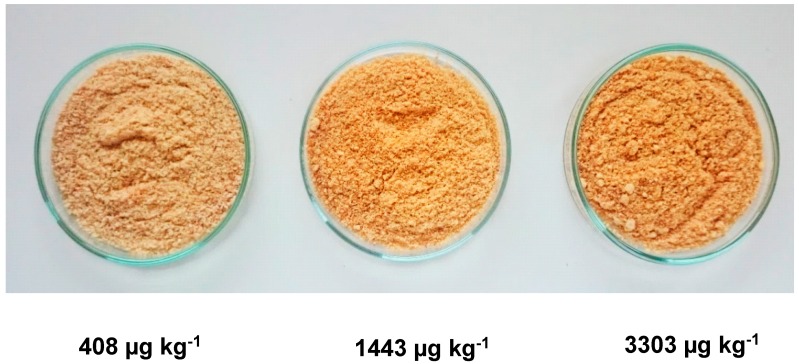
Photograph of the same lot of biscuits (Biscuit D) subject to different cooking temperatures. The different colors can be observed (Δ*L* = 5.53 and 2.25; Δ*a* = 3.61 and 0.61), as well as the corresponding acrylamide levels (ΔAA = 1035 and 1860 µg kg^−1^).

The AA content has been compared with both the colour, by measuring the Hunter Scale parameters, L, a and b in a colorimeter, as well as with the moisture content of biscuits collected in different oven positions. As can be seen in the images shown in Figure 3, the browning of the biscuit associated with the increase of the AA content is clearly observed. The Hunter colour scale parameters L (light) and a (red) increased during cooking steps (ΔL = 5.53 and 2.25; Δa = 3.61 and 0.61) confirming that a correlation exists between the AA content and the browning of the biscuit.

Multivariate statistical analyses were carried out in order to define the parameters which most well correlate with the AA content (Table 3). The L parameter, which is luminosity, is the first variable correlated with AA content whereas an inverse correlation was found between the moisture and the AA content. This is not surprising, since the temperature of the baking oven is expected to be negatively correlated with the final moisture content of the biscuit, showing the direct impact of temperature on the AA content. The influence of temperature on the formation of AA thus seems confirmed, as demonstrated in previous studies [13,49,50]. In addition, Jozinovic et al. [51] have recently shown that the moisture content and temperature during extrusion had a greater impact on the formation of AA in relation to screw speed. Recent results revealed that at low temperatures used for the thermal treatment, the amount of AA formed was lower, even if the treatment duration was longer [52]. In the current work, the baking times are identical for the four biscuit types, thus it is not possible to associate them with the AA values analyzed.

## 4. Conclusions

A sensitive and efficient HRMS methodology, based on LC-MS with electrospray ionization and Orbitrap as mass analyser, allowing quantification of AA for specific food matrices of biscuits was presented. Combining the multiplexed targeted-SIM mode for AA and isotopically labeled internal standard (acrylamide-d3), the proposed HRMS method enables reliable and accurate analyses of AA with very little influence by the matrix components. Under these conditions 3.55 µg kg^−1^ for LOD and 11.8 µg kg^−1^ for LOQ are attainable.

During baking an increase in AA concentration was observed, as well as for samples taken from the middle of the oven, where the temperatures are higher. Statistical processing of data shows that the composition of the raw materials of the biscuits was statistically the most correlated factor with the AA content. Statistical treatment shows the direct impact of temperature on the AA content as well.

This study also reported that two types of biscuits (out of four) contain AA in concentration clearly above the indicative value reported by European Food Safe Authority, confirming the pressure of establishing mitigation measures for the reduction of the presence of AA in these matrices.

## Figures and Tables

**Table 1:** Repeatability, limit of detection (LOD), and limit of quantification (LOQ) of the proposed methodology, based on several measurements of Biscuit A.

Assay	Acrylamide (AA) Content (µg kg^−1^)	Average AA Content (µg kg^−1^)	SD	RSD_r_ %	LOD (µg kg^−1^)	LOQ (µg kg^−1^)
1	254.1					
2	277.3					
3	343.9					
4	309.8					
5	269.8	297.9	33.1	11.1	3.55	11.8
6	345.0					
7	290.9					
8	292.0					

**Table 2:** Acrylamide content (µg kg^−1^) in biscuits collected from different points of the baking oven.

Biscuit	Edges of the Baking Oven (µg kg^−1^)	Middle of the Baking Oven (µg kg^−1^)	Average (µg kg^−1^)
A	216	431	324 ± 36
B	563	551	557 ± 61
C	1881	2231	2056 ± 226
D	1443	3303	2373 ± 261

**Table 3:** Correlation matrix between the acrylamide content and the colour and moisture of biscuits (Biscuit D). In bold, significant values (except diagonal) at the level of significance 95%.

	AA Content	L	a	b	Moisture
**AA content**	1.0				
**L**	**0.541**	1.0			
**a**	0.310	0.352	1.0		
**b**	0.276	**0.864**	0.663	1.0	
**Moisture**	**−0.277**	0.307	0.304	0.576	1.0

## Supplementary Materials

The following are available online at https://www.mdpi.com/2304-8158/8/12/597/s1, Figure S1: Chromatograms for a biscuit sample, indicating the acrylamide (AA) and deuterated acrylamide (AA-d3) retention times.
