# Supplementary figures and images for: Determination of Acrylamide in Biscuits by High-Resolution Orbitrap Mass Spectrometry: A Novel Application
Source: Foods. 2019 Nov 20;8(12):597. doi: 10.3390/foods8120597 (PMC6963597; doi:10.3390/foods8120597)

RT: 0,00 - 30,01 SM: 7B

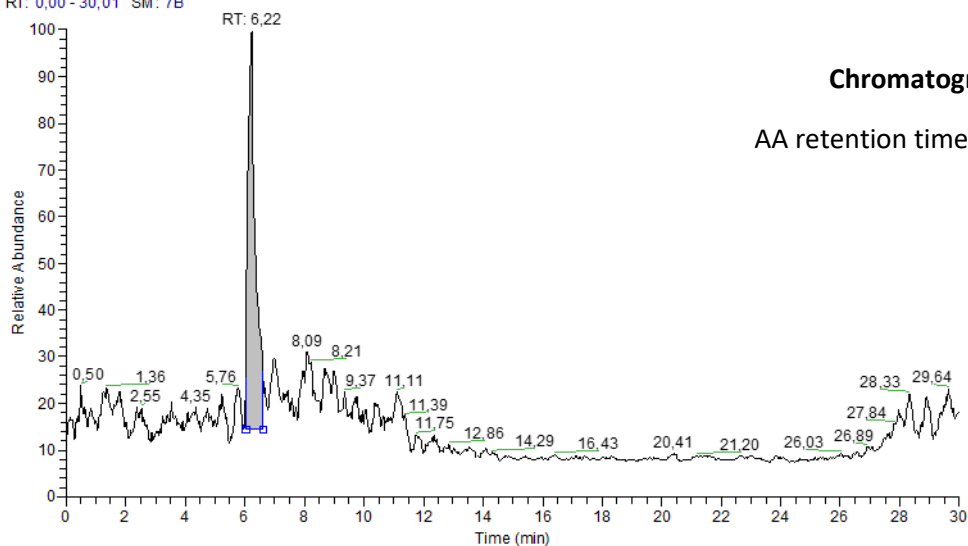

**Chromatogram A**

AA retention time: 6.22 min

RT: 1,56 - 15,60 SM: 7B

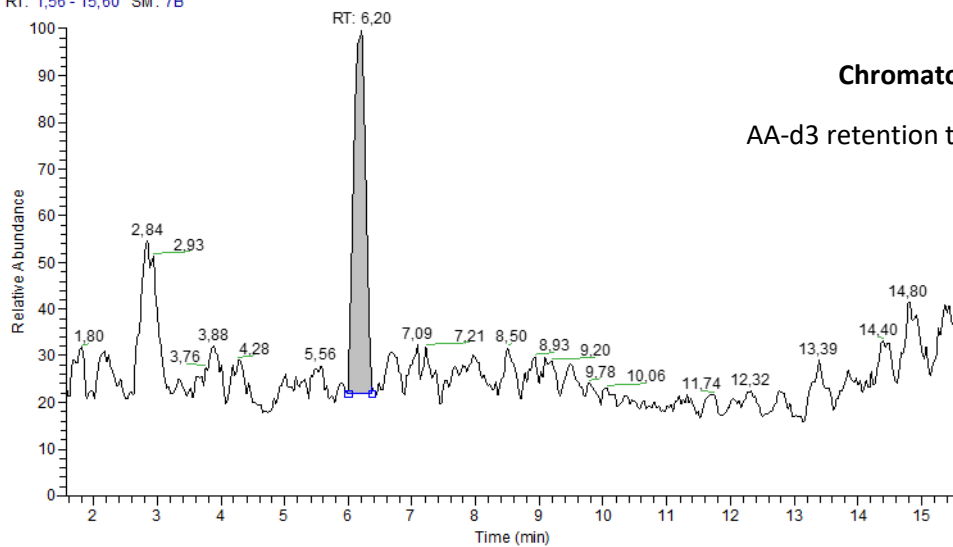

**Chromatogram B**

AA-d3 retention time: 6.20 min

Supplement: Supplementary file 1 [file foods-08-00597-s001.pdf]
